# Intense harvesting of eastern wolves facilitated hybridization with coyotes

**DOI:** 10.1002/ece3.61

**Published:** 2012-01

**Authors:** Linda Y Rutledge, Bradley N White, Jeffrey R Row, Brent R Patterson

**Affiliations:** 1 Biology Department, Trent University2140 East Bank Drive, Peterborough, Ontario, K9J 7B8, Canada; 2 Wildlife Research & Development Section, Ontario Ministry of Natural Resources, Trent University2140 East Bank Drive, Peterborough, Ontario, K9J 7B8, Canada

**Keywords:** Conservation, culling, demographic history, historic DNA, hybridization, lethal methods

## Abstract

Despite ethical arguments against lethal control of wildlife populations, culling is routinely used for the management of predators, invasive or pest species, and infectious diseases. Here, we demonstrate that culling of wildlife can have unforeseen impacts that can be detrimental to future conservation efforts. Specifically, we analyzed genetic data from eastern wolves (*Canis lycaon*) sampled in Algonquin Provincial Park (APP), Ontario, Canada from 1964 to 2007. Research culls in 1964 and 1965 killed the majority of wolves within a study region of APP, accounting for approximately 36% of the park's wolf population at a time when coyotes were colonizing the region. The culls were followed by a significant decrease in an eastern wolf mitochondrial DNA (mtDNA) haplotype (C1) in the Park's wolf population, as well as an increase in coyote mitochondrial and nuclear DNA. The introgression of nuclear DNA from coyotes, however, appears to have been curtailed by legislation that extended wolf protection outside park boundaries in 2001, although eastern wolf mtDNA haplotype C1 continued to decline and is now rare within the park population. We conclude that the wolf culls transformed the genetic composition of this unique eastern wolf population by facilitating coyote introgression. These results demonstrate that intense localized harvest of a seemingly abundant species can lead to unexpected hybridization events that encumber future conservation efforts. Ultimately, researchers need to contemplate not only the ethics of research methods, but also that future implications may be obscured by gaps in our current scientific understanding.

## Introduction

Although lethal sampling of wildlife for ecological experimentation was common up until the second half of the 20th century, the emergence of a stronger environmental ethic in recent decades has rendered the practice generally indefensible (Farnsworth and Rosovsky 1993; Minteer and Collins 2005; Vucetich and Nelson 2007). Culling of wildlife as a management tool, however, is routinely used to (1) increase the population size of desirable game species (Thirgood et al. 2001; Boertje et al. 2010; Schneider et al. 2010); (2) protect vulnerable endemic or domestic species from predators (Conner et al. 2008; Smith et al. 2010) or invasive exotics (Genovesi 2005); (3) impede disease transmission (Wasserberg et al. 2009; Lachish et al. 2010), or (4) acquire basic ecological knowledge for establishing sustainable harvest quotas (Morishita 2006) or effective conservation (Sillett et al. 2004). These methods are usually controversial, sprouting passionate counter arguments based on scientific and ethical considerations (e.g., Minteer and Collins 2005; Clapham et al. 2007; Vucetich and Nelson 2007).

The influence of human activities on the evolutionary trajectory of wildlife is widespread (see the January 2008 Special Issue of Molecular Ecology). Altered landscapes, climate change, invasive species, and direct harvest are shaping the genetic potential of species worldwide (Smith and Bernatchez 2008). In recent years, the impact of human-caused mortality on the genetic composition of populations has received much attention because exploitation fosters evolutionary alterations that may increase the risk of extinction (Stockwell et al. 2003; Burney and Flannery 2005), induce rapid evolution of life-history traits (Coltman et al. 2003; Allendorf and Hard 2009; Darimont et al. 2009), increase hybridization (Rhymer and Simberloff 1996), and impact behavioral dynamics in kin-based social groups (Gobush et al. 2008; Rutledge et al. 2010a). There is little doubt that intense harvest, especially over long time periods, results in genetic alterations that can be detrimental to populations and ecosystems (Allendorf et al. 2008). For example, when barriers to gene flow break down, genetic changes can result from hybridization between rare endemic and closely related invasive species, thereby impeding implementation of effective conservation policy (Allendorf et al. 2001), and increasing risk of extinction (Rhymer and Simberloff 1996). Although genetic effects of harvesting on wildlife are becoming well documented, the long-term impact that culling of seemingly abundant species has on genetic structure and conservation of populations is rarely considered.

Molecular genetic monitoring of populations over time is a powerful approach to facilitate an understanding of genetic changes in populations impacted by harvesting, particularly for small populations of threatened species (Allendorf et al. 2008; Coltman 2008). Interpreting genetic data within the context of demographic history is also critical to accurately explain genetic change (e.g., Jackson et al. 2008). Wolves across North America have been subjected to intense eradication efforts that have limited their genetic variability and evolutionary potential (Leonard et al. 2005), promoted coyote (*C. latrans*) expansion eastward (see Rutledge et al. 2010b), and increased coyote hybridization with eastern wolves (*C. lycaon*) (Kays et al. 2010; Way et al. 2010) and red wolves (*C. rufus*) (Fredrickson and Hedrick 2006; note that *C. lycaon* and *C. rufus* are suggested as the same species by Wilson et al. 2000).

**Figure 1:**
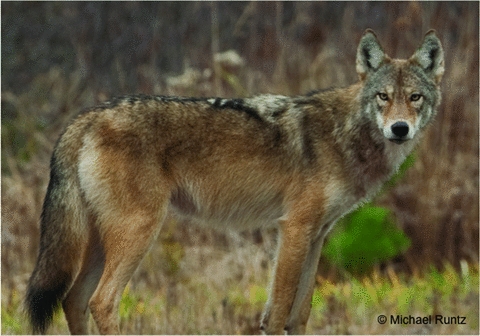
Eastern wolf (*Canis Lycaon*) photographed at Brule Lake in Algonquin Provincial Park. Photograph by Michael Runtz used with permission.

Seemingly limited to regions in and around Algonquin Provincial Park (APP; Rutledge et al. 2010c), eastern wolves (Fig. 1) are particularly susceptible to hybridization because of their shared evolutionary history with coyotes in North America (Wilson et al. 2000; Rutledge et al. 2010b) and their ability to bridge gene flow between gray wolves and coyotes (Rutledge et al. 2010c). In addition, eradication efforts over the past 400 years have substantially reduced the population size of eastern wolves (Boitani 2003), making them particularly susceptible to introgression from expanding coyotes due to an absence of suitable mates and the tendency for genes to flow asymmetrically from the more abundant into the more rare species (Grant et al. 2005). Patterns of introgression associated with human-caused reduction in population size have been noted in red wolves that hybridize extensively with coyotes (Fredrickson and Hedrick 2006) and Vancouver Island gray wolves that have introgressed dog genes (Muñoz–Fuentes et al. 2010).

Unlike gray wolves in the west, eastern wolves readily hybridize with coyotes (Rutledge et al. 2010c), and it has been suggested that high mortality of APP wolves could lead to gene swamping by coyotes (Theberge and Theberge 2004) that are ill-suited to occupy the niche of an apex predator and exert substantial top–down limitation of large ungulate prey species (i.e., deer and moose) due to their small size (e.g., Carbone et al. 1999). If intense harvesting of eastern wolves in APP results in increased hybridization with neighboring coyote populations, trophic interactions may be decoupled or otherwise altered. There has also been some suggestion that disruption to pack social structure associated with harvest pressure (Rutledge et al. 2010a) and breeder loss (Brainerd et al. 2008) could increase eastern wolf hybridization with coyotes when harvest occurs during breeding season.

**Figure 2:**
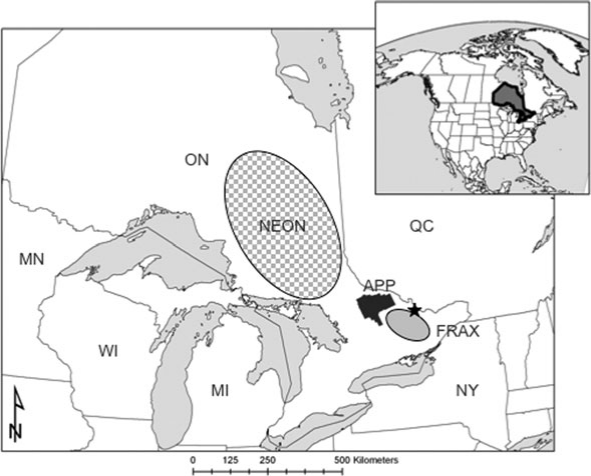
Map of Ontario, Canada. Dark gray area is Algonquin Provincial Park (APP) where samples were collected for this study over a 43-year period. Other samples used in this study include gray wolf–eastern wolf hybrid animals from northeastern Ontario (NEON; checkered oval) and coyote–eastern wolf hybrid animals from south of APP Park along the Frontenac Axis (FRAX; gray oval). Coyote population size indices for Figure 3 were taken from Wildlife Management Unit (WMU) 64B (black star).

Although wolves in APP, Ontario Canada (Fig. 2) are a morphologically and genetically differentiated group of approximately 200–300 eastern wolves that share a common evolutionary lineage with coyotes and red wolves (Wilson et al. 2000; Kyle et al. 2006, 2008; Rutledge et al. 2010b, d), prior to the year 2000, they were thought to be a gray wolf subspecies (*C. lupus lycaon*) that at the time was abundant across Ontario. Within the park, wolves have survived a long history of control efforts dating back to the park's establishment in 1893. Prior to the mid-1960s, wolves were actively poisoned, snared, and shot by park rangers in an effort to bolster game populations. Between 1909 and 1958, an average of 49 wolves per year (range 11–128) were killed in APP (Pimlott et al. 1969). In 1959, harvesting ceased within the park so that researchers could study an unexploited population of wolves. To conclude that study, researchers culled 80 wolves in 1964 and another 26 in 1965 in an effort to understand the reproduction and age structure of the population (Pimlott et al. 1969). The harvested wolves constituted the majority of wolves within the study area (population size estimate for the 2849 km^2^ study area was 90–110; Pimlott et al. 1969) and accounted for approximately 36% of the park's wolf population at the time (population size estimate for the total park [7725 km^2^] = 1 wolf/26 km^2^ = 297 wolves [Pimlott et al. 1969]). Since the end of the research project in 1965, wolves have been protected within the park, although human-caused mortality of migratory park animals still accounted for ∼60% of all wolf mortality in the eastern half of the park (Forbes and Theberge 1996; Theberge and Theberge 2004) until December 2001 when wolf protection was extended to all townships surrounding the park (Rutledge et al. 2010a).

**Figure 3:**
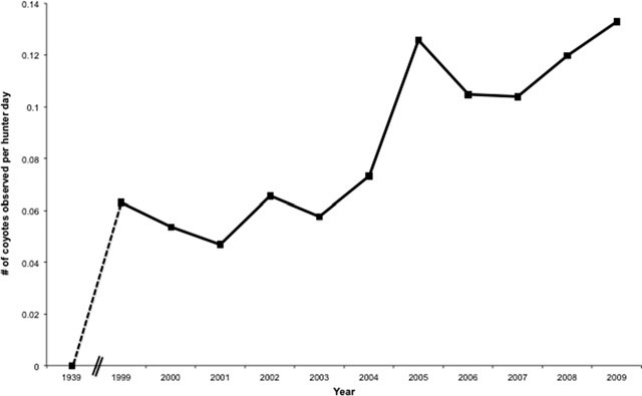
Trend in coyote observations in the Ottawa Valley region over the past 70 years. Value for 1939 is based on no coyotes reported in the Ottawa Valley prior to 1940 (Pimlott 1961). Data from 1999 to 2009 is calculated from coyote observations in Wildlife Management Unit (WMU) 64B of the Ottawa Valley reported by deer hunters on posthunt report cards. Dashed line and double slash indicate period of missing data.

Although wolf harvest in the first half of the 20th century presumably impacted the population size and altered the original genetic makeup of wolves within the park, the timing of the research culls in the mid-1960s is important because it occurred at a time when coyotes were becoming well established in the area. Prior to the 1960s, introgression from coyotes may have occurred, but was likely limited because the first coyote confirmed in southern Ontario was recorded in Thedford, Lambton County in 1919 (Nowak 1979) and densities near APP would have been relatively low until the beginning of the 1960s when coyote populations expanded rapidly north, east, and south (Moore and Parker 1992) in response to new habitat made available through land clearing and wolf extirpation (Kyle et al. 2006; Kays et al. 2010). Estimates of coyote abundance in Wildlife Management Unit 64B (Fig. 2) southeast of APP suggest a trend of increased density (Fig. 3). Therefore, there was presumably limited potential for coyote introgression into APP wolves during the first half of the 20th century, although immigration of wolf-like animals, either gray wolf–eastern wolf hybrids from northeastern Ontario or other Algonquin-type animals living in the park periphery, was likely common at the time. To explore the long-term impacts that wildlife culls can have on conservation, we analyzed genetic data acquired from eastern wolf samples collected in APP over a 43-year period (1964–2007), and interpreted genetic changes within the context of wolf and coyote demographic history in and around APP. Ultimately, this research demonstrates that although intense localized killing of an apparently abundant species may seem innocuous under the accepted scientific framework of the time, it may have lasting, and unforeseen, conservation implications.

## Methods

### Sample collection and DNA extraction

APP wolf samples used in this study were collected over 43 years in the different time periods: 1964–1965 (hereafter referred to as Historic Harvested [HH64–65]), 1987–1999 (hereafter referred to as Contemporary Harvested [CH87–99]), and 2002–2007 (hereafter referred to as Contemporary Protected [CP02–07]). Details regarding sample collection and DNA extractions for the CH87–99 samples can be found in Grewal et al. (2004) and for CP02–07 details are provided in Rutledge et al. (2010c). For HH64–65 samples, DNA was extracted from teeth samples removed from 40 skulls of adult and yearling wolves trapped and killed in APP during 1964 and 1965 (Pimlott et al. 1969). Given that boiling water maceration was used to clean these skulls, we attempted to extract DNA from the dried blood found inside intact canines and molars to improve the probability of obtaining larger fragments of DNA. Sample processing and DNA extractions were carried out in a laboratory area dedicated to the extraction of low-template DNA from historic and ancient samples at Trent University. The ancient DNA laboratory enforces strict protocols to minimize risk of contamination from contemporary sources. Filter tips or disposable transfer pipettes were used throughout the extraction process, and multiple negative controls were used to track reagent contamination.

Exterior surfaces of the teeth were decontaminated with a 1:9 DECON solution (Fisher Scientific, Ottawa, ON) to remove any foreign DNA and then rinsed with DNAase-free water (Gibco, Invitrogen, Burlington, ON). Teeth were crushed with a hammer to expose the inner vasculature and the dried blood from inside each tooth was placed in 400-µl 1× lysis buffer (4 M urea, 0.2 M NaCl, 0.5% *n*-lauroyl sarcosine, 10 mM CDTA [1, 2-cyclohexanediamine], 0.1 M Tris-HCl, pH 8.0) and incubated at 37°C overnight (12–18 h). Then 50 µl of Proteinase K (600 mAU/mL) was added to each sample followed by incubation at 55°C overnight with rotation. Samples were then stored at 37°C up to 2 days to ensure complete digestion. Samples were extracted by standard phenol–chloroform methods adjusted for small volumes (Sambrook and Russell 2001). Extracts were then concentrated over Amicon Ultra 0.5 mL Centrifugal Filters (Millipore, Billerica, MA) and stored at –20°C until amplified with polymerase chain reaction (PCR).

### DNA quantification, amplification, sequencing, and genotyping

Details regarding samples from CH87–99 and CP02–07 can be found in Grewal et al. (2004) and Rutledge et al. (2010c), respectively. HH64–65 samples were quantified by amplification of microsatellite primer cxx172 with PCR conditions described in Rutledge et al. (2010c) and 2 µl of DNA extract. To minimize effects of PCR inhibitors, 0.2 µg of bovine serum albumin (BSA) was added to all reactions. In addition, 1.5 Units of *Taq* DNA polymerase (Invitrogen) were added to each reaction to account for 35 PCR cycles. Amplified product was visualized on an ethidium bromide stained agarose gel, and fluorescence was compared to a positive control with 500 pg of DNA in the reaction with the software Quantity One (Bio-Rad, Mississauga, ON) to ensure that samples used in subsequent microsatellite reactions had at least 500 pg of DNA in each reaction and alleviate scoring errors due to allelic dropout (Rutledge et al. 2009 and references therein). The control sample was prepared outside the ancient DNA laboratory and added to the PCR machine immediately prior to the start of the reaction process. We followed this protocol for positive controls for all reactions so that amplification could be tracked, but risk of contamination was minimized. At all times during amplification and analysis, the positive control was handled after all other samples had been processed. For those samples where at least 500 pg of DNA could be put into a PCR, a multiplex reaction of 35 cycles with microsatellite primers cxx253, cxx147, cxx410, cxx442 and simplex reactions with microsatellite primers cxx225 and cxx172 were run to acquire individual genotypes. Reaction conditions and primer references are described in Rutledge et al. (2010c). For direct comparison, DNA from the CH87–99 wolf samples were amplified at these same six microsatellite loci and similarly scored.

For HH64–65 males (as identified in field notes) with sufficient target DNA, four Y chromosome microsatellite regions were amplified with primers MS34A, MS34B, MS41A, and MS41B (Sundqvist et al. 2001) with 40 cycles under conditions described in Rutledge et al. (2010c). DNA from the PCR product was precipitated with a standard ethanol precipitation and labeled fragments were separated on an AB3730 (Applied Biosystems, Carlsbad, CA). All autosomal and Y chromosome alleles were scored in GeneMarker 7.1 (SoftGenetics, State College, PA) and checked manually according to strict internal standards of peak height and morphology.

A 343- to 347-bp fragment of the mitochondrial DNA (mtDNA) control region was amplified from 2 ul of stock DNA with primers AB13279 and AB13280 (Wilson et al. 2000) under the following conditions: initial denaturation at 94°C for 5 min followed by 40 cycles of 94°C for 30 sec, 60°C for 30 sec, 72°C for 30 sec. Final extension was at 72°C for 2 min followed by storage at 4°C. Amplified product was visualized on an ethidium bromide stained agarose gel and samples with sufficient DNA were prepared with Exonuclease 1 (M0293S) and Anarctic Phosphatase (M0289S) (New England BioLabs Inc., Ipswich, MA) followed by sequencing with a Big Dye Terminator Kit (Applied Biosystems) in both forward and reverse directions on an AB3730. Consensus sequences of 343 bp were generated from contigs assembled from forward and reverse sequences in Sequencher 4.9 (GeneCodes Corporation, Ann Arbor, MI). All sequences were checked manually to ensure accurate base calling by the software.

### Analyses

Mitochondrial DNA and Y microsatellite haplotypes were assigned based on previously published nomenclature (Wilson et al. 2000; Rutledge et al. 2010c) and compared to previously published data for the CH87–99 (Grewal et al. 2004), and CP02–07 (Rutledge et al. 2010c). Due to widespread hybridization between eastern wolves and coyotes, it is difficult to make species designations to some haplotypes. Where there is discrepancy in the literature, both potential species origins are listed (for further discussion see Wheeldon et al. 2010; Rutledge et al. 2010c). To determine if the proportion of eastern wolf haplotype C1 had decreased in APP since the mid-1960s, we performed randomization tests of 1000 iterations with replacement in the statistical software package R 2.9.0 based on 23 sampling events of C1 from the CH87–99 and CP02–07 datasets.

Only those samples from the mid-1960s that had sufficient target DNA and amplified at four or more loci (*n* = 17) were used in subsequent microsatellite analyses. Data included in microsatellite analyses include those generated here (HH64–65 and CH87–99) as well as previously published data from CP02–07, gray wolf–eastern wolf hybrids from northeastern Ontario (NEON), and eastern coyotes from southern Ontario along the Frontenac Axis (FRAX) (see Rutledge et al. 2010c). In the HH64–65 dataset, 23% of samples had missing allele scores at cxx442 and 35% had missing allele scores at cxx147. Combined, 23% of samples had missing scores at both loci. To identify the impact of including loci with missing data in estimates of differentiation, we graphed *F*_st_ and Jost's *D*_est_ measures of genetic differentiation of the three APP time periods (HH64–65, CH87–99, and CP02–07) and NEON to FRAX at all six loci, then excluding cxx147 (five loci), and finally excluding locus cxx147 and cxx442 (four loci; Appendix A1). Trends were similar for all comparisons although including all six loci in some cases gave slightly more conservative estimates of differentiation. Therefore, we included all loci in subsequent analyses.

Measures of observed and expected heterozygosity, number of alleles, and private alleles were calculated in GenAlEx 6.3 (Peakall and Smouse 2006), as were standard measures of genetic distance (*F*_st_) and tests of significant differences between populations based on 999 permutations. Jost's *D*_est_ (Jost 2008) was also calculated in SMOGD (Crawford 2010; accessed June 22, 2010) because *F*_st_ values do not always reflect true differentiation based on shared alleles (Jost 2008). To assess changes in nuclear gene flow over time between APP animals and those of NEON and FRAX, we (1) assessed *F*_st_ and Jost's *D*_est_ comparisons, (2) conducted Bayesian clustering analysis in Structure 2.2 (Falush et al. 2007), (3) used principal components analysis (PCA) in R 2.9.0, and (4) implemented a logistic regression analysis in R 2.9.0. Details regarding determination of the number of clusters and the parameter settings for the Structure analysis, as well as PCA analysis of the microsatellite dataset are described in Rutledge et al. (2010c). In general, the number of clusters (*K*) in Structure was determined by assessing a plot of the log probability of the data (Mean LnP(*K*)) and a plot of the second-order rate of change of the likelihood function (Δ*K*) (Evanno et al. 2005) such that they were congruent with biological meaning. For the Structure analysis, we estimated the number of clusters with no a priori assignment under the F model for correlated allele frequencies with 5,000,000 MCMC steps and a burn-in of 250,000 for five runs each of *K* = 1–8. Subsequent to optimal *K* determination, we conducted 10 runs for *K* = 3 and averaged assignment scores (*Q*) (which represent the posterior probability of membership to each cluster) over the 10 runs. PCA was conducted in the adegenet package (Jombart 2008) of R (R Development Core Team 2008). For the logistic regression analysis, coyote-influenced animals (as described below) were coded as “1” and eastern wolf animals were coded as “0” to determine changes in coyote influence in APP during the three time periods. Similarly, in a separate logistic regression to determine changes in gray wolf influence, gray wolf animals were coded as “1” and eastern wolves were coded as “0” to determine changes in gray wolf influence in APP (comparing influence in mid-1960s to that of 2000s since there was no gray wolf influence noted in the 1980/90s). We identified an animal as a coyote-influenced animal if *Q*_FRAX_≥ 0.2 and a gray wolf influenced animal if *Q*_NEON_≥ 0.2 (based on the understanding that a first-generation hybrid backcrossed to a “pure” strain would result in an assignment score of 0.75, and on a hybrid simulation based power analyses for our ability to detect hybrids implemented in the adegenet package [Jombart 2008] in R 2.9.0 [unpublished data]). Hybrid influence scores were assigned as the dependent variable and the time period was assigned as the independent variable with HH64–65 as the reference dataset. *Q*-values distributed across all three groups were only found in CP02–07 (*n* = 12) and these samples were excluded from the logistic regression analysis because assignment scores split across all populations can be an indication that the source population has not been sampled rather than representing influence from all populations.

### Simulations

Coalescent simulations generate the genomes of individuals, moving backwards in time, under a defined demographic scenario with the assumption that the coalescent process (Kingman 1982) for neutral markers will be determined by the population and demographic history. Using coalescent simulations, one can determine the distribution of genetic summary statistics under a given demographic scenario and determine if the observed data fall within or outside of the expected distribution (e.g., Gray et al. 2008; Banks et al. 2010). In our analysis, an alternate explanation for the unexpected change in differentiation between eastern wolves in APP and coyotes in FRAX is genetic drift acting between sampling periods, rather than the impacts of harvesting. We therefore used coalescent simulations to establish a distribution of expected change in differentiation between APP wolves and FRAX coyotes through time under a demographic model, which does not include any impacts of the harvest. If the observed patterns were outside of this distribution, it is probable that genetic drift alone is not responsible for the observed patterns.

**Figure 4:**
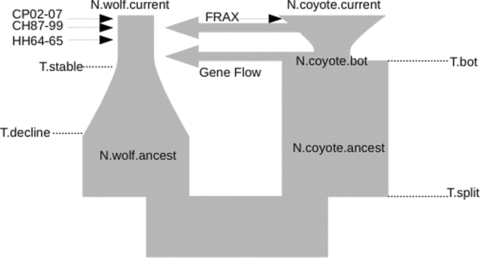
Assumed model of population and demographic history for eastern wolves and coyotes in eastern North America. See Table 1 for parameter estimates and text for description of the model.

Under our demographic model (Table 1; Fig. 4), eastern wolves and coyotes split between 150,000 and 300,000 years ago (T.split) (Wilson et al. 2000) and were separated until 100 years ago when the first coyotes were reported in southern Ontario (Nowak 1979). Separately, eastern wolves remained at a constant population size (N.wolf.anscest) of 64,500–90,200 individuals (estimated by multiplying the historic range throughout the eastern temperate forests (2,578,425 km^2^; CEC 1997) by an estimated density of eastern wolves (0.025–0.035/km^2^; Rutledge et al. 2010a) until 250–500 years in the past (T.decline) when European settlers came to North America and eastern wolf populations started to decline toward their current estimated population size (N.wolf.current) in and around APP of 500–1000 individuals (this value includes the Park population estimate of 300 [Rutledge et al. 2010a] plus individuals that occur outside of the park boundaries). The ancestral coyote population size (N.coyote.ancest) of 956,800–1,453,000 (estimated by multiplying the size of their historic range in the Great Plains [3,543,875 km^2^; CEC 1997] by an estimated density of coyotes [0.27–0.41/km^2^; Berger and Conner 2008]) was also modeled to remain stable until 100 years ago (T.bot) when a small number of individuals (Kays et al. 2010) (N.coyote.bot; estimated at 50–100) founded the population in southern Ontario and expanded to their current estimated size (N.coyote.current) of 45,600–69,300 (estimated by multiplying estimated coyote density by the size of the Mixed Woods Plains ecoregion in Ontario and Quebec [168,913 km^2^; Wiersma 2007]). After this founding population arrived, we allowed constant asymmetric gene flow (0.1–5%) from coyotes in FRAX into wolves in APP. For the model parameters, we estimated effective population sizes by dividing the estimated population size by average pack size (wolves = 5 [Loveless 2010]; coyotes = 4 [Way 2003]) and multiplying by two breeders per pack (Table 1). We assumed a strict stepwise mutation model with a mutation rate varying between 1.1 × 10^–2^ and 3.9 × 10^–3^ based on *Canis* microsatellite mutation rate estimates (Parra et al. 2010).

**Table 1:** Coalescent simulation parameters

Parameters	Min	Max
N. wolf.current	200	400
N.coyote.current	22,800	34,600
N.coyote.bot	25	50
N.wolf.ancest	25,800	36,080
N.coyote.ancest	478,000	726,000
T.stable	50	100
T.decline	250	500
T.bot	100	100
T.split	150,000	300,000
*Gene flow	0.001	0.05
Mutation	1.1 × 10^–3^	3.9 × 10^–3^

* Proportion of wolf population coming from coyote population. Population range estimates used in coalescent simulations aimed at modeling the demographic history of eastern wolves and coyotes. Population size values (*N*) are effective population sizes, and time values (*T*) are in years (number of generations × 5-year generation time). All parameter estimates varied within a uniform distribution. See text and Figure 1 for details of the demographic model.

The coalescent simulations were generated with Serial SimCoal (Anderson et al. 2005) within ABCtoolbox (Wegmann et al. 2010), which was used to vary the demographic parameters. Because Serial SimCoal allows for populations to be sampled at various time periods, we sampled the simulated wolf population (based on the midpoint of the sampling period) at 40 years in the past (HH64–65), 10 years in the past (CH87–99), and the current generation (CP02–07), and calculated *D*_est_ (Jost, 2008) between each of these samples and a sample from the simulated coyote population (*D*_est_ 1, *D*_est_ 2, and *D*_est_ 3, respectively). Sample sizes were consistent with observed data and *D*_est_ was calculated with a modified python script of SMOGD version 1.2.5 (Crawford, 2010). We wanted to determine if the change in differentiation was different than expected under the assumed demographic scenario, so we calculated the relative change in difference from HH64–65 to CH87–99 (Δ*D*_a_) as


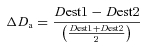


and the relative change in differentiation from CH87–99 to CP02–07 (Δ*D*_b_) as


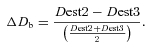


A value of 0 represents no change in differentiation; values > 0 suggests a decrease through time and values < 0 suggest an increase through time. Subsequently, we compared the observed relative change to the distribution produced from the 10,000 simulations to determine if the observed change was likely in the absence of harvest pressure.

## Results

The frequencies of mtDNA haplotypes changed over time (Table 2). Comparison of mtDNA haplotype names to those found in other studies is provided in Appendix A2. Randomization tests indicate that there was a significant decrease in the proportion of C1 eastern wolf haplotypes since the mid-1960s (HH64–65 mean = 0.478; CH87–99 mean = 0.119, SD = 0.065; CP02–07 mean = 0.0238, SD = 0.032). We were only able to obtain complete Y microsatellite profiles for two animals sampled from the mid-1960s, and both had eastern wolf haplotype AA (Table 2). Partial profiles were determined for nine other animals from the mid-1960s: seven had haplotype A for locus MS34, one had haplotype A for MS41, and one only amplified at one locus that was consistent with a probable A haplotype for MS34 (Appendix A3). Based on known Y chromosome haplotypes (Wilson et al. In Review), there are only three possible haplotypes for these partial profiles: AA, AQ, or EA (see Appendix A3). Since neither AQ, which occurs in Nebraska coyotes, nor EA, which occurs in Texas coyotes, are known to occur in Ontario (Wilson et al. In Review), it is likely that at least 10 of the 11 animals profiled have an eastern wolf haplotype AA. Given the high proportion of missing genotypes, however, we did not pursue further analysis or interpretation of the Y microsatellite data.

**Table 2:** Mitochondrial DNA (mtDNA) and Y microsatellite haplotypes from Algonquin Provincial Park (APP) during the three different sampling periods

mtDNA haplotypes				

Haplotype	Species affiliation	HH64–65	CH87–99	CP02–07
C1	Eastern Wolf	11	12	3
C3	Eastern Wolf	0	0	1
C13	Eastern Wolf	1	5	1
C17	Eastern Wolf	1	9	8
C22	Gray Wolf	0	4	9
C14	Coyote	9	35	65
C19	Coyote	0	18	33
C16	Coyote	0	1	0
C9	Coyote/Eastern Wolf*	1	18	7
Total (*n*)		23	102	127

Y microsatellite haplotypes				

Haplotype	Species affiliation	HH64–65	CH87–99	CP02–07
AA	Eastern Wolf	2	30	26
BB	Eastern Wolf	0	13	14
CC	Gray Wolf	0	2	0
CD	Coyote/Eastern Wolf*	0	4	2
CE	Gray Wolf	0	1	2
DC	Gray Wolf	0	1	0
EF	Gray Wolf	0	1	3
CR	Coyote/Eastern Wolf*	0	0	1
GP	Coyote/Eastern Wolf*	0	0	1
Total (*n*)		2	52	49

Data for Contemporary Harvested 1937–1999 (CH37–99) are from Grewal et al. (2004) and data for Contemporary Protected 2002–2007 (CP02–07) are from Rutledge et al. (2010b). Randomization tests (see text) indicate values of the eastern wolf mtDNA haplotype C1 are significantly lower in the 1980/90s (CH87–99) and 2000s (CP02–07) than in the mid-1960s (HH64–65). Sample size is small for the HH64–65 Y microsatellites due to difficulty in amplifying the regions on these histonic samples. Additional partial Y microsatellite profiles for the HH64–65 time period are available In Appendix A3.

* Widespread hybridization between western coyotes and eastern wolves has resulted in uncertainty regarding the species affiliation of these haplotypes. For a discussion see Wheeldon et al. (2010) and Rutledge et al. (2010b).

Heterozygosity in APP was high across all three time periods and was similar to surrounding regions; the number of effective alleles was also similar across time periods and populations (Table 3). Both *F*_st_ and Jost's *D*_est_ values showed the closest relationship between coyotes in FRAX and eastern wolves in APP occurred during the 1980/90s, whereas in the mid-1960s these two populations were more differentiated; differentiation increased from the 1980/90s to the 2000s but did not reach mid-1960s values (Table 4).

**Table 3:** Comparison of genetic diversity among populations

Population	Sample size (*n*)	*H*_o_ (SE)	*H*_e_ (SE)	*N*_a_ (SE)	*N*_e_ (SE)
NEON	51	0.686 (0.054)	0.628 (0.045)	5.667 (0.558)	3.021 (0.566)
HH64–65	17	0.693 (0.078)	0.678 (0.027)	4.833 (0.401)	3.214 (0.251)
CH87–99	41	0.748 (0.024)	0.727 (0.012)	7.000 (0.683)	3.695 (0.154)
CP02–07	128	0.672 (0.026)	0.722 (0.026)	7.000 (0.730)	3.753 (0.331)
FRAX	38	0.763 (0.048)	0.755 (0.030)	6.167 (0.543)	4.385 (0.525)

*H*_o_ = observed heterozygosity, *H*_e_ = expected heterozygosity, *N*_a_ = number of alleles, *N*_e_ = number of effective alleles, SE = standard error, NEON = northeastern Ontario, HH64–65 = Historic Harvested samples collected in Algonquin Provincial Park (APP) between 1964 and 1965, CH87–99 = Contemporary Harvested samples collected in APP between 1987 and 1999, CP = Contemporary Protected samples collected in APP between 2002 and 2007, FRAX = Frontenac Axis. Values are based on six microsatellite loci.

**Table 4:** Genetic distance between populations

Population	NEON	HH64–65	CH87–99	CP02–07	FRAX
Four loci					
NEON	n/a	0.403	0.264	0.298	0.354
HH64–65	0.166 (0.001)	n/a	0.010	0.002	0.160
CH87–99	0.130 (0.001)	0.012 (0.073)	n/a	0.006	0.078
CP02–07	0.125 (0.001)	0.007 (0.130)	0.003 (0.170)	n/a	0.159
FRAX	0.154 (0.001)	0.066 (0.001)	0.041 (0.001)	0.058 (0.001)	n/a
Five loci					
NEON	n/a	0.232	0.229	0.269	0.330
HH64–65	0.145 (0.001)	n/a	0.022	0.012	0.165
CH87–99	0.119 (0.001)	0.024 (0.007)	n/a	0.001	0.057
CP02–07	0.117 (0.001)	0.022 (0.002)	0.002 (0.241)	n/a	0.130
FRAX	0.145 (0.001)	0.071 (0.001)	0.035 (0.001)	0.051 (0.001)	n/a
Six loci					
NEON	n/a	0.246	0.239	0.269	0.274
HH64–65	0.161 (0.001)	n/a	0.028	0.020	0.149
CH87–99	0.124 (0.001)	0.039 (0.001)	n/a	0.000	0.047
CP02–07	0.118 (0.001)	0.035 (0.001)	0.001 (0.308)	n/a	0.112
FRAX	0.138 (0.001)	0.080 (0.001)	0.032 (0.001)	0.047 (0.001)	n/a

Values are based on 4, 5, or 6 autosomal micosatellite loci. *F*_st_ is below horizontal and Jost's *D*_est_ is above horizontal. *P*-values for *F*_st_ comparisons (in parentheses) are based on 999 permutations in the AMOVA option of GenAlEx. NEON = northeastern Ontario, HH64–65 = Historic Harvested samples collected in Algonquin Provincial Park (APP) between 1964 and 1965, CH87–99 = Contemporary Harvested samples collected in APP between 1987 and 1999, CP = Contemporary Protected samples collected n APP between 2002 and 2007, FRAX = Frontenac Axis.

**Figure 5:**
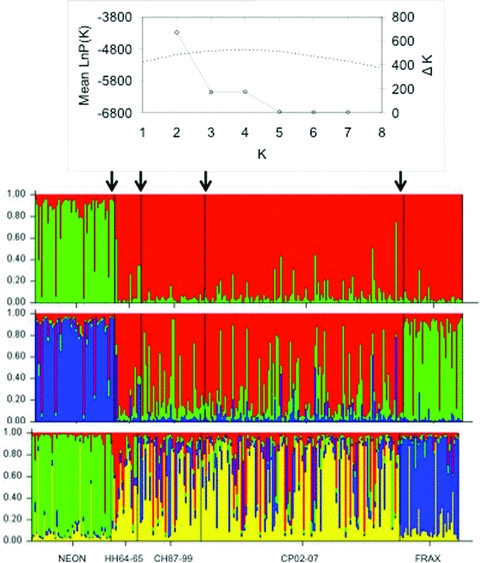
Number of *Canis* clusters inferred from six autosomal microsatellites. Top figure shows mean log probability of the data (dashed line) and the second-order change of the likelihood function (Δ*K*) (solid line) as a means of inferring the number of clusters in the data. Arrows indicate “population” divisions, APP = Algonquin Provincial Park. At *K* = 2, the major division between Old World evolved animals (gray wolves) and New World evolved animals (eastern wolves and coyotes) occurs. At *K* = 3, eastern coyotes separate and APP animals from all three time periods cluster together. *K* = 4 hints at a division within Algonquin animals, but this division is difficult to interpret biologically and should be treated with caution. Overall, *K* = 3 is the most likely number of clusters.

**Figure 6:**
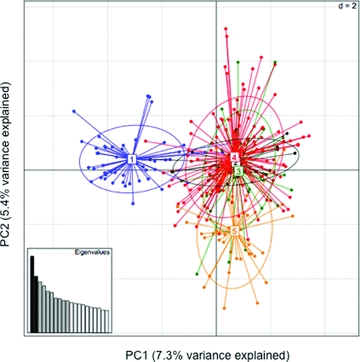
Centered, scaled PCA analysis of six autosomal microsatellite loci from the five different groups. Population 1 in blue = northeastern Ontario (NEON), 2 in black = Algonquin Provincial Park (APP) from mid-1960s (Historic Harvested 1964–1965 [HH64–65]), 3 in green = APP from the 1980/90s (Contemporary Harvested 1987–1999 [CH87–99]), 4 in red = APP from the 2000s (Contemporary Protected 2002–2007 [CP02–07]), and 5 in orange = Frontenac Axis (FRAX).

Analysis of the autosomal microsatellite data with Structure and PCA identified three main clusters in the dataset, with the three APP clusters having overlapping profiles (Figs. 5 and 6), although the HH64–65 data were more tightly clustered in the PCA (Fig. 6). As in other analyses of similar datasets (e.g., Rutledge et al. 2010c), the Δ*K* peak at *K* = 2 represents the major division between Eurasian-evolved (Old World) gray wolves and North American-evolved (New World) species. The high Δ*K* values at *K* = 3 and *K* = 4 provide more subtle clustering information of more recently diverged groups. As shown in Figure 5, *K* = 4 is not biologically informative, thus *K* = 3 is suggested as the optimal number of clusters for this dataset.

**Figure 7:**
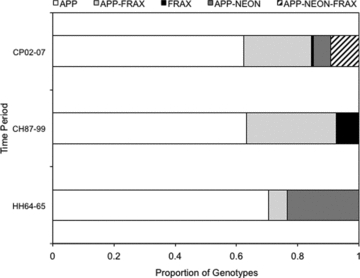
Proportional representation of wolves in APP in the three different time periods assigned in Structure as (A) Algonquin Provincial Park (APP; *Q* ≥ 0.8 to APP); (B) influenced by hybridization with eastern coyotes from Frontenac Axis (APP-FRAX; 0.8 ≥ *Q* ≥ 0.2 to FRAX); (C) strongly assigned to FRAX (FRAX; *Q* ≥ 0.8 to FRAX); (D) influenced by hybridization with gray wolf–eastern wolf hybrids from northeastern Ontario (APP-NEON; 0.8 ≥ *Q* ≥ 0.2 to NEON); (E) assigned with *Q* ≥ 0.2 to all three populations (APP-NEON-FRAX). HH64–65 = Historic Harvested samples collected between 1964 and 1965; CH87–99 = Contemporary Harvested samples collected between 1987 and 1999; CP = Contemporary Protected sampled collected between 2002 and 2007.

Differences among the three Algonquin datasets were not readily obvious from these analyses. Results of the logistic regression, however, indicate a significant increase in the proportion of coyote-like animals in APP from the mid-1960s to the 1980/90s (parameter estimate = 2.223; SE = 1.081; df = 2, 171; *P* = 0.0397) but not from the mid-1960s to the 2000s (parameter estimate = 1.674; SE = 1.053; df = 2, 171; *P* = 0.112) (Fig. 7). Odds of finding a coyote-like animal were 9.1 times higher in the CH87–99 dataset than HH64–65, but only 5.3 times higher in the CP02–07 data. In contrast, there was a significant decrease in the number of gray wolf influenced animals in the park over time. In the CH87–99 dataset, there were no animals sampled with genetic influence from NEON and logistic regression of the HH64–65 compared to the CP02–07 suggest a significant decrease (parameter estimate = –1.567; SE = 0.692; df = 1; *P* = 0.0236). Odds of sampling a gray wolf influenced animal were reduced by a factor of 0.21 in CP02–07 compared to HH64–65.

### Simulations

**Figure 8:**
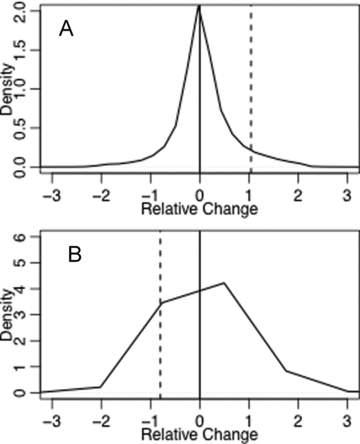
Kernel density plots of the relative change in Jost *D*_est_ between coyotes in the Frontenac Axis (FRAX) and wolves in APP from, (A) Historic Harvested 1964–1965 (HH64–65) to Contemporary Harvested 1987–1999 (CH87–99) and (B) CH87–99 to Contemporary Protected 2002–2007 (CP02–07), for 10,000 coalescent simulations (see text for details of simulations). Values of 0 represent no change in differentiation, values > 0 represent a decrease, and values < 0 represent an increase. Dotted vertical line shows the observed change.

The observed relative change in population differentiation between HH64–65 and CH87–99 (Δ*D*_a_) was 1.04, which was within the range, but greater than 93% (*P* = 0.06) of the coalescent simulations (Fig. 8), suggesting that differentiation between coyotes and wolves decreased more than expected under the defined demographic model. Conversely, the observed relative change between CH87–99 and CP02–07 (Δ*D*_b_) was –0.80 and lower than 95% (*P* = 0.05) of the simulations, suggesting the observed magnitude of gene flow from FRAX to APP was smaller than expected under constant migration across time periods.

## Discussion

Killing of wolves during the mid-1960s in APP appears to have influenced the genetic composition of the Park's wolf population. Although researchers at the time could not have predicted these outcomes, it seems likely that extensive culling of wolves prompted the few remaining wolves in the Park to mate with individuals from the expanding coyote population. The subsequent decline of an eastern wolf mtDNA haplotype and introgression of coyote mitochondrial and nuclear DNA correlates well with the demographic history of the two species, and coalescent simulations suggest these outcomes were unlikely in the absence of harvest pressure. The genetic consequences of this hybridization have complicated eastern wolf conservation and may continue to do so in regions where APP wolves disperse into unprotected areas where coyotes flourish (e.g., Quebec and southern Ontario).

The exact impacts and biological mechanisms of the mtDNA exchange are unclear, but a similar turnover of mtDNA haplotypes associated human-caused gray wolf extirpation followed by recolonization and subsequent dog introgression has been noted in Vancouver Island gray wolves (Muñoz–Fuentes et al. 2010). Similar to the situation on Vancouver Island, hybridization between eastern wolves and coyotes in APP may have occurred due to an Allee effect (Allee 1931) resulting from a lack of conspecific mates for eastern wolves associated with small population size when wolf harvest was high. Like the situation on Vancouver Island, maintaining large population sizes and minimizing human-caused mortality will be important for minimizing potentially deleterious effects of hybridization. For eastern wolves in APP, affording protection for wolves in connected, suitable eastern wolf habitat between the Park and surrounding regions will be important for promoting gene flow among eastern wolves that will maximize genetic variability on which natural selection can act. Although nuclear genetic diversity of APP wolves was maintained over time, their nuclear genetic signature is now closer to the mid-1960s state than it was in the 1980/90s when park animals were genetically more similar to eastern coyotes. We attribute this genetic restoration to the implementation of a ban in 2001 on wolf hunting and trapping in the townships surrounding the park where high human-caused wolf mortality occurred for wolves migrating outside park boundaries (Forbes and Theberge 1996; Theberge and Theberge 2004). Thus, expanded protection may have promoted the natural recovery of a historic genetic state. This rebound is important because genetic influence from the smaller coyote may be detrimental to the viability of the wolf population in the current park ecosystem where moose are the most common ungulate prey (Quinn 2004; Loveless 2010), and larger body size is positively related to predatory efficiency when hunting large ungulates (Carbone et al. 1999; MacNulty et al. 2009).

We have shown that intensive eastern wolf culls may exacerbate hybridization with coyotes. These results may have implications for other closely related species that have been brought together by landscape changes and expansion of nonendemics. Wolves have been extirpated across most of their original range in North America with dramatic consequences for wolf viability and ecosystem health. For example, extirpation has led to widespread loss of genetic diversity within wolf populations thus reducing their adaptive evolutionary potential (Leonard et al. 2005), and ecosystems have suffered considerably in the absence of top predators that effectively regulate ungulate populations (Beschta and Ripple 2009; Licht et al. 2010). The impacts of overharvesting are widespread across species. It is a global problem that has left small, remnant populations of amphibians, birds, mammals, and fish susceptible to extinction through hybridization with closely related, more abundant, invasive species (Rhymer and Simberloff 1996). In the face of increasing habitat alteration, invasion of nonendemic species, and climate change, the mapping of evolutionary processes over time is of utmost importance for wildlife conservation (Smith and Bernatchez 2008). As demonstrated here, utilizing historic samples for long-term genetic monitoring of populations is essential for tracking changes in the evolutionary trajectory of a population and implementing effective conservation and management strategies, especially for exploited populations (Allendorf et al. 2008; Coltman 2008; Darimont et al. 2009).

Above all, our results demonstrate that intense localized harvesting of species thought to be numerous and widespread can have unexpected outcomes that threaten conservation of species and naturally functioning ecosystems. The advanced molecular genetic techniques now used for studying wildlife populations were unheard of in the 1960s and no one could have predicted the impacts that such an experimental design could have on a population. Although the research methods used in the 1960s would fail to meet current ethical guidelines, targeted culling is still common practice for managing wildlife under various scenarios (Genovesi 2005; Karki et al. 2007; Wasserberg et al. 2009; Lachish et al. 2010; Smith et al. 2010). For example, lethal control of gray wolves (*C. lupus*) is currently used to increase the size of ungulate populations in Alaska, USA (Boertje et al. 2010), and in Alberta, Canada (Schneider et al. 2010) where both total wolf harvest and areas of intense harvest (>45 wolves/1000 km^2^) have increased over the past 22 years (Robichaud and Boyce 2010). Similarly, lethal methods are routinely used for coyote control, with intense “spatially clumped” harvest suggested as more effective than random removal across a broad spatial scale (Conner et al. 2008). Coyotes are generally regarded as vermin, and wolves are often perceived as a major threat to ungulate populations; both of these viewpoints were similarly applied toward wolves in APP prior to 1965.

Our results suggest the potential for ecological assumptions to be incomplete and that culling and other seemingly harmless, invasive methods, even when applied to abundant “pest” species, may have unexpected, lasting conservation implications. Whether for the purpose of game species management, protection of endemics, population size estimates, or collecting basic ecological knowledge, exploring nonlethal alternatives could minimize unanticipated impacts to animal populations and thus reduce the burden on wildlife managers. By following guidelines and principles of ecological ethics as outlined by a growing number of scientists (Farnsworth and Rosovsky 1993; Minteer and Collins 2005; Vucetich and Nelson 2007; Paquet and Darimont 2010), sampling methods are less likely to result in unanticipated negative impacts. In this way, we can avoid leaving behind a legacy of complications for future conservation biologists and wildlife managers.

## Figures and Tables

**Appendix A1:**
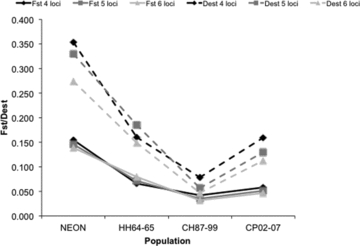
Comparison of genetic distance values (F_st_ and D_est_) based on 4. 5. and 6 microsatellite loci. All populations are compared to eastern coyotes from the Frontenac Axis (FRAX). Data were complete for 4 loci of all populations, but for 5 loci, HH64–65 was missing 23% of data at locus cxx442 and for 6 loci it was also missing 35% at locus cxx147. NEON = gray-eastem wolf hyorids from northeastern Ontario; HH64–65 = Historic Harvested sampled in Algonquin Provincial Park (APP) between 1964–55: CHB7–99 = Conternporary Harvested sampled in APP between 1987–99: CP02–07 = Contemporary Protected sampled between 2002–07. Data for FRAX, NEON and CP02–07 are from Rutledge *et al.* (2010c).

## Appendix

**Appendix A2:** Comparison of haplotypes with the published litrature. (a) Leonard & Wayne 2008; (b) Koblmüller et al. (2009); (c) Wilson *et al.* (2000); (d) Hailer & Leonard (2008); (e) Rutledge *et al.* (2010d)

Sample ID	Sequence length (bp)	mtDNA Haplotypes
6345/64	343	GL10, GL17, GL18^a^; C13^c^; Ccr10^e^
6242/65	343	GL12^a^; C17^e^; Ccr11^e^
6347/64	343	GL13^a^; Ia33^d^; C14^c^; Ccr09^e^
6342/64	343	GL13^a^; Ia33^d^; C14^c^; Ccr09^e^
6244/65	343	GL13^a^; Ia33^d^; C14^c^; Ccr09^e^
6257/65	343	GL13^a^; la33^d^; C14^c^; Ccr09^e^
6288/65	343	GL13^a^; Ia33^d^; C14^c^; Ccr09^e^
6252/65	343	GL13^a^; Ia33^d^; C14^c^; Ccr09^e^
6254/65	343	GL13^a^; Ia33^d^; C14^c^; Ccr09^e^
6250/65	343	GL13^a^; Ia33^d^; C14^c^; Ccr09^e^
6290/64	343	GL13^a^; Ia33^d^; C14^c^; Ccr09^e^
6246/65	342	GL16^a^; Ia18^?^; C9^c^; Ccr29^e^
6352/64	343	GL1^a^; Ia19^b^; C1^c^; Ccr12^e^
6315/64	343	GL1^a^; Ia19^b^; C1^c^; Ccr12^e^
6240/65	343	GL1^a^; Ia19^b^; C1^c^; Ccr12^e^
6307/64	343	GL1^a^; Ia19^b^; C1^c^; Ccr12^e^
6311/64	343	GL1^a^; Ia19^b^; C1^c^; Ccr12^e^
6256/65	343	GL1^a^; Ia19^b^; C1^c^; Ccr12^e^
6253/65	343	GL1^a^; Ia19^b^; C1^c^; Ccr12^e^
6241/65	343	GL1^a^; Ia19^b^; C1^c^; Ccr12^e^
6283/65	343	GL1^a^; Ia19^b^; C1^c^; Ccr12^e^
6346/64	343	GL1^a^; Ia19^b^; C1^c^; Ccr12^e^
6269/64	299	GL1^a^; Ia19^b^; C1^c^; Ccr12^e^

**Appendix A3:** Y-microsatellite scores for Historic Harvested samples collected between 1964–65 (HH64–65), Haplotypes are assigned based on previously published literature (see Rutledge et al. 2010b). Several values are missing due to the difficulty in amplifying MS41 in these historic samples. However, the only known haplotype combinations where values are missing are indicated. It is probable that MS41 haplotypes with missing alleles (marked *) are A haplotypes because the other possibility is AQ (Wilson et al. In Review) that occurs in Nebraska coyotes and is not known to occur in Ontario. Similarly, it is probably that the one ^**^ is A because the only other known alternative is E which occurs in Texas coyotes and red wolves (Wilson et al. In Review) and is not known to occur in Ontario

Sample			MS34			MS41	Y
ID	MS34A	MS34B	Haplotype	MS41A	MS41B	Hapotype	Haplotype
6240/65	172	180	A	212	212	A	AA
6285/65	172	180	A	212		A, Q*	A?
6244/65	172	180	A	212	**−**	A, Q*	A?
6307/64	172	**−**	−	**−**	**−**	**−**	?
6311/64	172	180	A	**−**	**−**	**−**	A?
6309/64	172	180	A	**−**	**−**	−	A?
6253/65	172	180	A	**−**	**−**	**−**	A?
6241/65	**−**	**−**	A, E**	212	212	A	?A
6252/65	172	180	A	212	**−**	A, Q*	A?
6242/65	172	180	A	**−**	**−**	**−**	A?
6283/65	172	180	A	212	212	A	AA
